# Antibodies to biotin enable large-scale detection of biotinylation sites on proteins

**DOI:** 10.1038/nmeth.4465

**Published:** 2017-10-16

**Authors:** Namrata D Udeshi, Kayvon Pedram, Tanya Svinkina, Shaunt Fereshetian, Samuel A Myers, Ozan Aygun, Karsten Krug, Karl Clauser, Dominic Ryan, Tslil Ast, Vamsi K Mootha, Alice Y Ting, Steven A Carr

**Affiliations:** 1 Proteomics, Broad Institute of MIT and Harvard, Cambridge, Massachusetts, USA.; 2 Departments of Genetics, Biology, and Chemistry, Stanford University, Stanford, California, USA.; 3 DRI2 LLC, Littleton, Massachusetts, USA.; 4 Howard Hughes Medical Institute, Department of Molecular Biology, Massachusetts General Hospital, Boston, Massachusetts, USA.

## Abstract

Although purification of biotinylated molecules is highly efficient, identifying specific sites of biotinylation remains challenging. We show that anti-biotin antibodies enable unprecedented enrichment of biotinylated peptides from complex peptide mixtures. Live-cell proximity labeling using APEX peroxidase followed by anti-biotin enrichment and mass spectrometry yielded over 1,600 biotinylation sites on hundreds of proteins, an increase of more than 30-fold in the number of biotinylation sites identified compared to streptavidin-based enrichment of proteins.

The biotin–streptavidin system is a powerful and widely used tool in biotechnology. Chemical or enzymatic biotinylation of molecules is commonly employed for affinity purification, western blotting, immunohistochemistry, ELISA, and cell surface labeling. The remarkable strength of the noncovalent interaction between biotin and streptavidin (*K*_d_ ≈ 10^−14^ M) makes the system highly effective for purifying biotinylated molecules^1^. However, in analyses where it is necessary to identify the specific sites of biotinylation (e.g., S-nitrosylation^2^, sulfenylation^3^, and proximity labeling of proteins to define their subcellular location and topology^4^), the strength of the biotin–streptavidin interaction becomes a liability because harsh, denaturing conditions are necessary to break these interactions; and recovery of biotinylated molecules is poor. Alternate methods for eluting biotinylated proteins include competitive elution with free biotin under denaturing conditions, on-bead digestion of bound proteins^5^, modifying avidin molecules to have a lower affinity for biotin (NeutrAvidin or monomeric avidin), use of affinity-reducing biotin analogs such as desthiobiotin^6^, and implementation of cleavable biotin derivatives^7^. While these methods have been used together with liquid chromatography-mass spectrometry (LC-MS) for identification of biotinylated proteins, they have all fallen short of achieving reproducible, large-scale identification of biotinylation sites, especially as compared to the number of site identifications for other protein modifications, such as phosphorylation, ubiquitylation, and acetylation^8^.

We reasoned that antibodies directed against biotin could be used to enrich biotinylated peptides in a manner similar to that of the antibody-based enrichment of post-translational modifications^8^ (Fig. 1a). The binding affinity of anti-biotin antibodies for biotin may be weaker compared to avidin or streptavidin for biotin, and this could improve researchers’ ability to elute biotinylated peptides after affinity enrichment. As an initial test, we labeled peptides derived from whole-cell lysate digests with NHS-biotin (Supplementary Fig. 1a) and spiked the biotinylated peptide mixture at various levels into unlabeled peptide mixtures from the same whole-cell lysate digest. At a 1:50 ratio of biotinylated peptides to nonlabeled peptides, 129 biotinylated peptides were identified by LC-MS/MS analysis without enrichment (Supplementary Table 1). In contrast, enrichment with an anti-biotin antibody yielded 4,810 distinct biotinylated peptides on average from the 1:50 biotin peptide to nonbiotin peptide spike-in samples, and it yielded >3,000 distinct biotinylated peptides from the 1:2,000 biotin peptide to nonbiotin peptide spike-in samples (Supplementary Fig. 1b and Supplementary Table 1). Titration experiments using spike-in samples identified the optimal input of anti-biotin antibody as 50 μg for 1 mg peptide input (Supplementary Fig. 1c and Supplementary Table 1). We compared the performance of several commercially available anti-biotin antibodies and found the reagent from ImmuneChem Pharmaceuticals to yield the highest number of biotinylated peptides (Supplementary Fig. 1d). Using spike-in samples, we found that the enrichment of biotinylated peptides using our antibody-based approach was two- to three-fold higher than that of NeutrAvidin (Supplementary Table 2)^9^. Additionally, the antibody method was simpler to execute, as it has fewer sample-handling steps^9^.

We and others have implemented various peroxidase-mediated biotin-labeling methods in combination with quantitative MS to biotinylate, enrich, and identify proteins localized to particular subregions of the cell such as the mitochondrial matrix, the intermembrane space (IMS), the synaptic cleft, and cilia^10–14^. In previous work, we identified 495 mitochondrial matrix proteins using streptavidin-based protein enrichment, but we identified only 151 distinct biotinylation sites on 123 proteins^11^. Detection of biotinylation sites would provide direct evidence of proximity labeling, which could potentially provide additional information on protein topologies and could identify new subsets of proteins that were not previously identified using streptavidin enrichment.

We compared the number of biotinylated peptides identified by streptavidin- or antibody-based enrichment in proximity-labeled samples (Fig. 1b). APEX2 was targeted to the mitochondrial matrix (mito-APEX2) of SILAC-labeled HEK293T cells, and biotinylation was induced as previously described^4,11^. Streptavidin blot analysis of cell lysates showed that numerous proteins were biotinylated in an APEX2- and H_2_O_2_-dependent manner (Supplementary Fig. 2a). Imaging by confocal microscopy showed that biotinylated proteins overlapped tightly with the mito-APEX2 construct (Supplementary Fig. 2b).

Streptavidin-based enrichment from 2 mg of protein per SILAC channel followed by LC-MS (Fig. 1b) revealed successful labeling of mitochondrial matrix proteins using mito-APEX2 in live cells (Supplementary Table 3). We compared our enriched protein data to carefully curated lists of true-positive and false-positive proteins to select a SILAC ratio cutoff, as has been previously described (Supplementary Fig. 3)^4^. We identified 671 putative mitochondrial proteins in at least two replicates and 511 proteins in three replicates (Supplementary Tables 3 and 4). Only 185 distinct biotinylation sites were identified in total, of which 38 were detected in two or more replicates (Supplementary Table 5), which was consistent with our previous findings^4^. In sharp contrast, we identified 1,695 biotinylation sites using the antibody-based-enrichment workflow (Fig. 1b and Supplementary Table 6), 1,122 of which were observed in at least two replicates (Fig. 1c and Supplementary Table 7). Thus, 30-fold more biotinylation sites were reproducibly identified using antibody-based biotinylated peptide enrichment versus streptavidin-based biotinylated protein enrichment (Fig. 1c). The biotinylated peptides identified by antibody enrichment in at least two replicates derived from 526 proteins (Fig. 1c; Supplementary Tables 4 and 7). It is likely that we identify more biotinylated proteins using streptavidin-based protein enrichment versus antibody-based peptide enrichment because in the latter workflow, direct detection of biotinylated peptides is required for protein identification, while in the former workflow, any nonbiotinylated peptide meeting a statistical cutoff threshold is used for identification. Together, these two enrichment strategies provide highly complementary information—protein enrichment with streptavidin provides a larger list of potential APEX-labeled proteins, while immunoprecipitation (IP) with anti-biotin antibodies provides direct, higher confidence detection of labeled proteins, along with biotin-site identifications compared to streptavidin.

After oxidation of biotin-phenol by APEX2 in the presence of H_2_O_2_, the phenoxyl radical cannot diffuse out of the mitochondrial matrix^11^. To compare the mitochondrial specificity of our anti-biotin IP with streptavidin enrichment, we mapped biotinylated peptides or streptavidin-enriched proteins to a list of known mitochondrial proteins. For both enrichment strategies, we found that 80% of the proteins identified in common across three biological replicates were highly enriched for mitochondrial proteins, which indicated that there is no spatial bias between these enrichment methods (Supplementary Table 4). To characterize the relative coverage between the two enrichment strategies, we focused on the components of the oxidative phosphorylation (OXPHOS) system that are well characterized with respect to compartmental localization and membrane topology (Fig. 2a and Supplementary Table 8). Using streptavidin enrichment, we detected 22 OXPHOS proteins, all of which had at least part of their sequence exposed to the matrix space^11^. Using anti-biotin IPs, we observed biotinylated peptides for 17 of the 22 OXPHOS proteins identified using streptavidin enrichment, which demonstrated the specificity and membrane impermeability of the tagging^11^ (Fig. 2a and Supplementary Table 8). The direct detection of biotinylated peptides for OXPHOS proteins further supports the hypothesis that these proteins are specifically labeled by APEX2 and are not indirect interactors of proteins that are being biotinylated by proximity labeling. We did not detect biotinylation sites for ATP5D, NDUFV1, NDUFS2, NDUFS7, and UQCRFS1 (Fig. 2a and Supplementary Table 8), potentially because of insufficient sensitivity for these specific biotinylated peptides or because the biotinylated peptides are not amenable to detection by LC-MS/MS. Together these data suggest that the two enrichment strategies are comparable in terms of specificity and coverage.

The ability to identify large numbers of biotinylation sites provides essential data to understand the labeling characteristics of APEX2 biotinylation. Analysis of the large numbers of MS2 spectra for biotinylated peptides revealed product ions specific to biotin-phenol or biotinylated tyrosine^15^ that could be used to further increase detection of biotinylated peptides (Supplementary Fig. 4). Incorporating these signature product ions in the peptide spectral match scoring increased the number of biotinylated peptides by 11–12% (Supplementary Table 9). In addition, our data clearly establish tyrosine as the primary site of biotinylation by APEX2, with less than 2% labeling observed at alternate residues (tryptophan or cysteine) (Supplementary Fig. 5). Computational assessment of the surface exposure of these biotinylated tyrosine residues indicated that labeling occurred primarily at surface-exposed residues (see Online Methods).

Defining the exact sequence locations of biotinylation could provide direct insight into the topological organization of proteins in membranes. To this end, we identified 100 biotinylation sites on 46 mitochondrial transmembrane proteins in at least two replicates (Fig. 2b). 42 sites were identified on proteins with known membrane topology, 41 of which mapped to a matrix-exposed site on a transmembrane protein (Fig. 2b,c). MCU, the pore-forming subunit of the mitochondrial calcium uniporter (a calcium-activated calcium channel complex within the mitochondrial inner membrane^16^), was among the labeled transmembrane proteins; it had one modification near the N terminus (Y128) and one modification near the C terminus (Y299). The contentious question of MCU’s membrane topology^17,18^ was recently resolved by creating MCU-APEX chimeras for high-resolution electron microscopy (EM) imaging^19^; this showed that heterologously expressed MCU has an N-in, C-in topology. The site identification of endogenous MCU resulting from the anti-biotin antibody enrichment further corroborate the original finding^17^ that both the N and C termini of MCU are matrix facing, and serves as an example of the power of accurately identifying endogenous sites (Fig. 2c).

The only biotin site identified for a mitochondrial transmembrane protein with known topology that was not previously annotated to be matrix exposed was Y141 of LETM1 (Fig. 2c and Supplementary Table 7). Interestingly, Y141 resides at the N terminus of LETM1 and is reported to be facing the IMS^20^. IMS-exposed LETM1 is also consistent with our previous work, which used ratiometric tagging with APEX to map the proteome of the IMS^10^. These findings suggest two possibilities: either LETM1 is present in two subpopulations, which have different membrane topologies or LETM1 undergoes conservative sorting into the mitochondrial inner membrane. Proteins sorted in the IMS via the conservative sorting pathway translocate to the matrix before their insertion in the intermembrane^21,22^. An enrichment of proline residues in the transmembrane domain has been shown to drive the conservative sorting mechanism^23^, and indeed LETM1 has three conserved proline residues in its predicted transmembrane domain^22^, which strengthens this hypothesis. We propose that LETM1 has an N-out, C-in topology with its predicted EF hands facing the mitochondrial matrix (Supplementary Fig. 6). To our knowledge, LETM1 is the only mitochondrial protein whose EF hand resides within the matrix.

Here we describe a robust and sensitive workflow for enrichment and identification of biotinylated peptides using an anti-biotin antibody. The lack of reliable methods for enrichment of biotinylated peptides rather than biotinylated proteins has hampered the detection of biotinylation sites from biological samples. For robust detection of post-translational modifications from complex mixtures, it is usually necessary to implement an affinity-enrichment step for modified peptides, as has been shown for other protein modifications^8^. The major advantage of enriching peptides over proteins is that modified peptides can be separated from nonmodified peptides which, when present, significantly reduce sensitivity for detection of less abundant modified peptides. Enrichment of intact biotinylated proteins would not likely improve detection of biotinylated peptides, as the overwhelming mass of peptides will consist of nonbiotinylated peptides derived from the biotinylated proteins as well as nonspecifically enriched, nonbiotinylated proteins.

Beyond applications in proximity labeling (APEX2 or BioID^14^), we expect that this workflow will dramatically improve detection of biotinylation sites in applications where avidin and/or streptavidin are currently used. We envision the use of anti-biotin antibodies for the analysis of protein membrane topology; cell-surface labeling sites; small molecule–protein interaction sites; protein crosslinking sites; mapping of sites of sulfenylation, palmitoylation, and S-nitrosylation; and for any other workflow for which biotin site mapping is necessary.

## METHODS

Methods, including statements of data availability and any associated accession codes and references, are available in the online version of the paper.

## ONLINE METHODS

A **Supplementary Protocol** describing the antibody-based biotinylated peptide enrichment workflow is available.

### Cell culture.

For label-free experiments, Jurkat E6–1 cells (ATCC) were grown in Roswell Park Memorial Institute 1640 (RPMI) media (Gibco) supplemented with 10% fetal bovine serum (FBS) (Gibco), penicillin, and streptomycin (Gibco). For SILAC experiments human embryonic kidney 293T (HEK293) cells transfected with APEX2 fusion construct targeted toward mitochondrial matrix and containing V5 epitope tag were grown in Dulbecco’s modified Eagle medium (DMEM) (Sigma) deficient in l-arginine and L-lysine and supplemented with 10% FBS (Gibco), penicillin, streptomycin (Gibco), and D-glucose (Sigma) (Hung *et al.*^10^). Cells were grown in SILAC media supplemented with either l-arginine (Arg 0) and L-lysine (Lys 0) or with l-arginine ^13^C_6_-^15^N_4_-HCL (Arg 10) and L-lysine ^13^C_6_-^15^N_2_-HCL (Lys 8).

### Biotin-phenol labeling.

Three biological replicates of SILAC-labeled HEK293T cells were labeled with biotin-phenol (BP) (Iris Biotech GmbH) at >80% confluency using previously described methods^4^. Specifically, cells were incubated with 25 ml of DMEM media containing 25 μl of 500 mM BP in DMSO (Sigma) for 30 min at 37 °C. Following incubation, heavy-isotope-labeled SILAC cells were treated with 1 mM hydrogen peroxide (H202) (Sigma) for 1 min at room temperature (RT). Treatment was omitted for light SILAC cells. The labeling solution was aspirated, and cells were washed three times with 25 ml of quencher solution (1× phosphate buffed saline (PBS) (Sigma), 10 mM sodium azide (Sigma), 10 mM sodium ascorbate (Sigma), and 5 mM Trolox (Sigma)). Following washes with the quencher solution, cells were washed 3× with 25 ml of 1× PBS. Cells were pelleted by centrifugation at 1,500× *g* for 10 min at 4 °C, snap frozen, and stored at −80 °C. Three replicate cell pellets were prepared for each SILAC state for both streptavidin-based protein enrichment and anti-biotin antibody enrichment experiments.

### Biotin spike-in experiments.

Jurkat cells were lysed with 8 M urea, 50 mM Tris-HCl pH 8.0, 150 mM NaCl, 1 mM EDTA (Sigma), 2 μg/μl Aprotinin (Sigma-Aldrich), 10 μg/μl Leupeptin (Roche), and 1 mM phenylmethylsulfonyl fluoride (PMSF) (Sigma) at 4 °C. Lysates were centrifuged at 20,000× *g* for 15 min at 4 °C to remove insoluble material. Protein concentration was determined using a bicinchoninic acid (BCA) protein assay (Pierce). Proteins were reduced with 5 mM dithiothreitol (DTT) for 45 min at RT and subsequently carbamidomethylated with 10 mM iodoacetamide for 30 min at RT in the dark. Prior to digestion, the urea concentration was reduced to 2 M with 50 mM Tris-HCl pH 8. Samples were predigested with Lysyl Endopeptidase (Wako Pure Chemical Industries) for 2 h at 30 °C at an enzyme:substrate ratio of 1:50. Sequencing-grade trypsin (Promega) was then added to samples at an enzyme:substrate ratio of 1:50, and the digestion was completed overnight at 25 °C.

### Peptide labeling with NHS-biotin.

Jurkat peptides were labeled with EZ link NHS-Biotin (Thermo Fisher Scientific). The biotin reagent was brought to RT. For labeling, 1 mg of peptide from Jurkat cell lysate was reconstituted in 1 ml of 50 mM HEPES (pH 8.5). 8 mg of biotin reagent was reconstituted in 410 μl of DMSO immediately before use and added directly to peptides. The biotin reagent and peptides were reacted for 30 min. Excess biotin in the reaction was quenched with 480 μl of 5% hydroxylamine for 15 min at RT while shaking. Following quenching, labeled peptides were dried to completion using vacuum centrifugation, resuspended in 5% acetonitrile/0.1% formic acid solution, and desalted using 500 mg SepPak columns (Waters). Desalted peptides were eluted using 50% acetonitrile/0.1% formic acid solution and dried down using vacuum centrifugation.

### Spike-in experiments and antibody titration curve.

A polyclonal anti-biotin (ImmuneChem Pharmaceuticals Inc., cat no. ICP0615) was used for biotin peptide enrichment. Peptide spike-in samples were composed of varying amounts of biotinylated peptides spiked in to nonbiotinylated peptides derived from Jurkat cells. Spike-in samples were created using the following ratios of biotinylated:nonbiotinylated peptides: 1:50; 1:100; 1:200; 1:1,000; 1:2,000. Enrichment of each sample was completed in triplicate using 1 mg of peptide input and 50 μg of anti-biotin antibody input. Specifically, peptides were reconstituted in 1 mL of 50 mM MOPS pH 7.2, 10 mM sodium phosphate, and 50 mM NaCl (IAP buffer). Anti-biotin antibody was washed 3× with IAP buffer and added to peptide samples. Samples were incubated with end-over-end rotation for 1 h at 4 °C. The solutions were spun down at 1,000× *g* for 1 min. The flowthrough was removed. Antibody beads were washed 4× with 1.5 ml of ice-cold PBS. Biotinylated peptides were eluted from the antibody with 50 μl of 0.15% TFA. Antibody beads were spun down at 1,000× *g* for 1 min, and the eluent was transferred to a new microfuge tube. This process was repeated, the eluents were combined, and the samples were desalted using StageTips exactly as previously described^25,26^. Briefly, each StageTip column was packed with two C18 Empore high-performance extraction disks (3M). Prior to sample loading, StageTips were conditioned with 100 μl of 100% MeOH, 100 μl of 50% MeCN, and 2× with 100 μl of 0.1% formic acid. Peptides were loaded onto conditioned StageTips, washed 2× with 100 μl of 0.1% FA, and eluted with 50 μl of 50% MeCN, 0.1% FA. Eluted peptides were dried by vacuum centrifugation.

For anti-biotin antibody titration curve experiments, the biotinylated:nonbiotinylated peptide ratio used was 1:1,000. Enrichment of samples for the anti-biotin antibody titration curve experiments was completed in duplicate using 1 mg peptide input and the following amounts of anti-biotin antibody: 25, 50, 100, 150, and 200 μg. Enrichment was completed exactly as described above.

Enrichment with anti-biotin antibodies was tested using antibody reagents from different vendors. Anti-biotin antibodies were tested from ImmuneChem (ICP0615), Sigma-Aldrich (A1559), and Santa Cruz (sc-101339). For these experiments, enrichments were completed exactly as described above using 30 μg of antibody input and 1 mg peptide input of 1:1,000 spike-in samples.

### Enrichment of biotinylated peptides using NeutrAvidin beads.

2 mg of dried peptide derived from Jurkat cells was resuspended in 1 ml of 1× PBS. Once peptides were fully dissolved, 2 μg of EZ-Link NHS-biotin-labeled peptide were spiked into the solution, which produced a 1:1,000 spike in. Neutravidin-based enrichment was completed exactly as previously described^9^. A 200 μl slurry of Pierce NeutrAvidin beads (Thermo Fisher Scientific) was added to the solution. The solution was left to incubate for 1 h at RT with end-over-end rotation. Following incubation, the solution was spun down at 1,000× *g* for 5 min. The flowthrough was collected and frozen. Beads were washed with 1 ml of 1× PBS (Gibco). The sample was spun down at 1,000× *g* for 1 min and the supernatant was aspirated. Beads were washed an additional two times with 1× PBS. Following three PBS washes, beads were washed 3× with 1 ml of 5% MeCN in PBS. Beads were then washed 3× with 1 ml of PBS. Lastly, beads were washed 3× with 1 ml of HPLC-grade water. There were a total of 12 washes performed to the beads. Biotinylated peptides were eluted by adding 300 μl 80% MeCN/0.1% formic acid/0.2% TFA solution to the beads. The first step of elution was completed with 300 μl of 80% MeCN/0.1% formic acid/0.2% TFA. The microfuge tube was gently tapped, spun down on a galaxy centrifuge at 1,000× *g*, and eluent was collected into a separate 15 ml tube. An additional 300 μl of 80% MeCN/0.1% formic acid/0.2% TFA solution was added to the beads. Beads were then boiled at 96 °C for 5 min. Beads were then spun down on a galaxy centrifuge at 1,000× *g*, and eluent was transferred to the original 15 ml tube. An additional 300 μl 80% MeCN/0.1% formic acid/0.2% TFA solution was added to the beads. Beads were gently tapped and spun down, and the eluent was transferred to the 15 ml tube. This step was completed an additional seven times. Ten total elution steps were completed. 3 ml of eluent was frozen and lyophilized. Dried peptides were resuspended in 0.1% formic acid and desalted on C18 stage tips exactly as described above. Samples were dried by vacuum centrifugation. Parallel enrichments of the same batch of 1:1,000 biotin peptide spike-in samples were completed using the anti-biotin antibody exactly as described above.

### Characterization of APEX2-mediated biotinylation by fluorescence imaging.

Labeling and imaging were carried out as previously described^4^. Human embryonic kidney (HEK) 293T cells stably expressing matrix-APEX2 (ref. 27) were grown on 7 × 7 mm glass coverslips to 60–80% confluence. Biotin-phenol was added in cell culture media to a final concentration of 500 μM, and the cells were incubated for 30 min at 37 °C, 5% CO_2_. H_2_O_2_ was added at a final concentration of 1 mM for 1 min. To quench the biotinylation reaction, the cells were washed three times with a solution of 10 mM sodium azide, 10 mM sodium ascorbate, and 5 mM Trolox in Dulbecco’s phosphate-buffered saline (DPBS). Cells were then fixed in 4% paraformaldehyde in PBS at room temperature for 15 min, washed three times with PBS, and permeabilized with prechilled methanol at −20 °C for 10 min.

Fixed cells were blocked for 1 h at room temperature with 3% BSA in PBS (‘blocking buffer’). To detect biotinylated proteins, cells were then treated with 50 nM NeutrAvidin-AlexaFluor(AF)647 in blocking buffer. NeutrAvidin-AF647 was prepared by coupling NeutrAvidin with AF647-NHS ester (Invitrogen) following the manufacturer’s instructions. APEX2 expression was detected by staining the fixed cells with mouse anti-V5 antibody (Life Technologies, Cat. No. R960–25; 1:1,000) in blocking buffer for 1 h at room temperature. Samples were then washed 4 × 5 min with PBS and incubated with goat anti-mouse-AF488 antibody (Thermo Fisher Scientific R37120; 1:1,000) in blocking buffer for 1 h at room temperature. Samples were washed 4 × 5 min with PBS then kept in DPBS at room temperature for imaging.

Confocal imaging was performed using a Zeiss AxioObserver. Z1 microscope with a Yokogawa spinning disk confocal head and a Cascade II:512 camera. The confocal head contained a Quad-band notch dichroic mirror (405/488/568/647 nm). Samples were excited by solid-state 491 (~20 mW) or 640 nm (~5 mW) lasers. Images were acquired using Slidebook 5.0 software (Intelligent Imaging Innovations), through a 63× oil-immersion objective for YFP/AF488 (528/38 emission filter), AF647 (700/75 emission filter), and differential interference contrast (DIC) channels. Imaging conditions and intensity scales were matched for images presented together.

### Sample preparation and streptavidin enrichment of proteins biotinylated by mito-APEX2.

Lysis and streptavidin enrichment was performed as previously described^4^. Briefly, SILAC-labeled cells were lysed with 1 ml of RIPA lysis buffer composed of 50 mM Tris-HCl, 150 mM NaCl, 0.1% SDS, 0.5% sodium deoxycholate, 1% NP40, and 1 mM EDTA, 1× protease inhibitor cocktail (Sigma-Aldrich), 1 mM PMSF, 5 mM Trolox, 10 mM sodium azide, and 10 mM sodium ascorbate. Lysates were incubated on ice for 4 min, followed by centrifugation at 15,000× *g* for 10 min at 4 °C. Protein concentration was determined for each sample using a Pierce 660 nm protein assay (Thermo Fisher Scientific). A total of 2 mg protein per SILAC state was used for proteomic experiments.

Pierce streptavidin magnetic beads (Thermo Fisher Scientific) were washed 2× with 1 ml of RIPA lysis buffer. Samples were incubated with 500 μl of streptavidin bead slurry overnight at 4 °C with gentle end-over-end rotation. Streptavidin beads were pelleted using a magnetic rack and the flow through was saved at −80 °C for quality control analysis. Pelleted beads were washed 2× with 1 ml of RIPA lysis buffer, followed by 1 ml of 1 M KCl, 1 ml of 0.1 M Na_2_CO_3_, 1 ml of 2 M urea in 10 mM Tris-HCl (pH 8) and 2× with 1 ml of RIPA lysis buffer. For each wash, all buffers were kept ice cold. Biotinylated proteins were eluted from streptavidin beads by boiling beads for 10 min at 96 °C in 60 μl of 3× NuPAGE LDS Sample Buffer (Invitrogen) with 2 mM biotin (Sigma) and 20 mM DTT.

### In-gel protein digestion.

Biotinylated proteins eluted from streptavidin beads were separated on a NuPAGE Novex Bis-Tris 4–12% gel (Thermo Fisher Scientific) for 1 h at 130 V as previously described^4^. Briefly, the gel was stained overnight with SimplyBlue Coomassie SafeStain (Thermo Fisher Scientific) and destained with water for several hours. Each gel lane was cut into 16 gel slices, and each slice was placed in a separate microfuge tube. Each slice was cut into small gel pieces. Gel pieces were washed 1× with 200 μl of 100 mM ammonimum bicarbonate (pH 8) and further destained overnight with 200 μl 50:50 MeCN:100 mM ammonium bicarbonate. Following this, gel pieces were washed with 100 μl of 100 mM ammonium bicarbonate and dehydrated for 5 min with 100 μl of 100% MeCN. Dehydrated gel pieces were incubated with 100 μl of 10 mM DTT in 100 mM ammonium bicarbonate for 1 h with shaking, followed by 100 μl of 55 mM iodoacetamide for 45 min in the dark, and then dehydrated with 100% acetonitrile. Digestion was completed by adding 10–50 μl of 10 ng/μl sequencing-grade Trypsin (Promega) to each gel sample and completed overnight at RT with shaking. Peptides were extracted by incubating gel pieces 3× in 60% MeCN:0.1% TFA solution followed by 1× incubation with 100% MeCN. The extraction solution was dried down by vacuum centrifugation. Samples were desalted exactly as described above using C18 StageTips.

### Anti-biotin antibody enrichment of peptides biotinylated by mito-APEX2.

SILAC-labeled cells were lysed with 8 M urea, 50 mM Tris-HCl pH 8.0, 150 mM NaCl, 1 mM EDTA, 2 μg/μl Aprotinin, 10 μg/μl Leupeptin, 1 mM PMSF (Sigma), 10 mM sodium azide, and 10 mM sodium ascorbate at 4 °C. Following lysis, cells were centrifuged at 20,000× *g* for 15 min at 4 °C to remove insoluble material. Protein concentration was determined using a BCA protein assay. SILAC-labeled samples were prepared by combining 2 mg of protein input per SILAC state. SILAC samples from three biological replicates were prepared. Proteins were reduced with 5 mM DTT for 45 min at RT and subsequently carbamidomethylated with 10 mM iodoacetamide for 30 min at RT in the dark. Prior to digestion, the urea concentration was reduced to 2 M with 50 mM Tris-HCl pH 8. Samples were predigested for 2 h at 30 °C with Lysyl Endopeptidase (Wako Pure Chemical Industries) at an enzyme:substrate ratio of 1:50. Samples were digested overnight at 25 °C with sequencing-grade trypsin at an enzyme:substrate ratio of 1:50.

Following digestion, samples were acidified with formic acid and desalted on a 500 mg tC18 Sep-Pak SPE cartridge (Waters). Cartridges were conditioned 1× with 5 ml of 100% MeCN, 1× with 5 ml 50% MeCN:0.1% FA, and 4× with 5 ml of 0.1% TFA. Samples were loaded, and cartridges were washed 3× with 5 ml of 0.1% TFA and then 1× with 5 ml of 1%FA. Peptides were eluted with 6 ml of 50%MeCN:0.1%FA, dried by vacuum centrifugation, and stored at −80 °C. SILAC samples were enriched using anti-biotin antibodies exactly as described above using 50 μg of antibody per immunoprecipitation (IP). Samples were desalted using C18 StageTips exactly as described above.

### Western blotting.

For western blot analysis, a small amount of sample was reserved from SILAC HEK293T cells labeled with biotin-phenol and mito-APEX from three replicates of both light and heavy conditions. Cells were lysed using RIPA lysis buffer, as described above, mixed with 3× LDS sample buffer containing 20 mM DTT, and separated on NuPAGE Novex Bis-Tris 4–12% gel. Proteins were transferred from gels to a nitrocellulose membrane using the iBLOT Dry Blotting System (Thermo Fisher Scientific) at 23 V for 6 min. The membrane was blocked with a mixture of PBS and 5% BSA for 1 h at RT, rinsed with PBS, and incubated overnight with a primary monoclonal mouse anti-V5 antibody (Invitrogen, cat. no. E10/V4RR) at 1:5,000 dilution in PBS with 5% BSA at 4 °C with orbital shaking. The blot was rinsed in PBST containing 0.1% BSA, and it was incubated with IRDye 800CW streptavidin antibody (Li-Cor, 925–32230 1:10,000 dilution) and with secondary IRDye 800CW anti-mouse antibody (Li-Cor, 925–32212, 1:10,000 dilution) for 1 h at RT while shaking. Following incubation, the blot was washed with 4× PBST and 2× in PBS, and it was imaged using an Odyssey Clx Li-COR system.

### Mass spectrometry analysis.

Peptides were reconstituted in 9 μl of 3% MeCN/0.1% FA and analyzed by online nanoflow LC-MS/MS using a Q Exactive Plus mass spectrometer (Thermo Fisher Scientific) coupled online to a Proxeon Easy-nLC 1200 or Proxeon Easy-nLC 1000 (Thermo Fisher Scientific). Briefly, 4 μl of sample was loaded onto a microcapillary column (360 μm OD × 75 μm ID) containing an integrated electrospray emitter tip (10 μm) (New Objective) packed with 24 cm of ReproSil-Pur C18-AQ 1.9 μm beads (Dr. Maisch GmbH). The nanoflow column was heated to 50 °C using a column heater (Pheonix S&T). NHS-labeled peptide spike-in samples and streptavidin-enriched samples were analyzed using a 110 min LC-MS method. Mobile phase flow rate was 200 nl/min. Solvent A was comprised of 3% acetonitrile/0.1% formic acid. Solvent B was comprised of 90% acetonitrile /0.1% formic acid. The 110 min LC-MS/MS method used the following gradient profile: (min:%B) 0:2; 1:6; 85:30; 94:60; 95:90; 100:90; 101:50; 110:50 (the last two steps at 500 nl/min flow rate). Anti-biotin-antibody-enriched APEX-labeled samples were analyzed using a 260 min LC-MS/MS method with the following gradient profile: (min:%B) 0:2; 1:6; 235:30; 244:60; 245:90; 250:90; 251:50; 260:50 (the last two steps at 500 nl/min flow rate). For all samples, MS1 spectra were measured with a resolution of 70,000, an AGC target of 3 × 106, and a mass range from 300 to 1,800 *m/z*. Up to 12 MS/MS spectra were triggered per duty cycle at a resolution of 17,500, an AGC target of 5× 104, an isolation window of 1.6 *m/z*, a maximum ion time of 120 ms, and a normalized collision energy of 25. The dynamic exclusion time was set to 20 s, the peptide match was set to preferred mode, and isotope-exclusion function was enabled. Charge state screening was enabled to reject precursor charge states that were unassigned, 1, or >6.

### Mass spectrometry data processing.

All data were analyzed using Spectrum Mill software package v 6.1 prerelease (Agilent Technologies). Similar MS/MS spectra acquired on the same precursor *m/z* within ±60 s were merged. MS/MS spectra were excluded from searching if they were not within the precursor MH+ range of 600–4,000 Da, or if they failed the quality filter by not having a sequence tag length >0. MS/MS spectra were searched against a UniProt database containing 59,079 human proteins. The database was downloaded from the UniProt website on October 17, 2014; redundant sequences were removed; and a set of common laboratory contaminant proteins (150 sequences) was appended. All spectra were allowed ±20 p.p.m. mass tolerance for precursor and product ions, 30% minimum matched peak intensity, and ‘trypsin allow P’ enzyme specificity with up to four missed cleavages. The fixed modification was carbamidomethylation at cysteine. For biotin spike-in data, allowed variable modifications were biotinylated (+226.0776 Da, +C10 H14 O2 N2 S1) peptide N termini and lysine, oxidized methionine, and acetylation of protein N termini with a precursor MH+ shift range of −18 to 550 Da. For SILAC data, arginine and lysine were searched as Arg 0–10 Da and Lys 0–8 Da -mix (R,K). Allowed variable modifications were acetylation of protein N termini, oxidized methionine, and biotin-phenol on tyrosine (+361.14601 Da, +C18 H23 N3 O3 S1) with a precursor MH+ shift range of −18 to 795 Da. The variable modifications of biotin and biotin-phenol were configured to account for marker ions resulting from fragmentation of the label to form immonium ion and related ions. These marker ions have the following *m/z* values and elemental composition losses from the fully modified amino acid. Biotin (K): 329.1 *m/z* (-C O). Biotin-phenol (Y): 497.22170 *m/z* (-C O); 480.19515 *m/z* (-C O N1 H3); 227.08487 *m/z* (-C17 H18 N2 O3). Identification of these diagnostic marker ions in the data set is described in the section below. For studying alternate sites of biotin-phenol labeling, SILAC data were processed as described, except that allowed variable modifications were carbamidomethylation at cysteine and biotin-phenol on tyrosine, tryptophan, and cysteine. Individual spectra were automatically assigned a confidence score using Spectrum Mill autovalidation module. A target–decoy FDR of 1% was used for biotin site data. For protein data, protein-polishing autovalidation was applied to further filter the peptide matches (PSMs) using a target-protein-level FDR threshold of zero.

### Identification of diagnostic marker ions.

Confidently assigned peptide-spectrum matches (PSMs) were analyzed for fragment ion masses that could not be assigned to canonical peptide fragment ion types during MS/MS database search. To that end, peak lists (*m/z*, intensity – pairs) for each PSM were generated using the Ion Assignments tool part of Spectrum Mill MS Proteomics Workbench. For each peptide spectrum, the tool extracted the 25 most abundant MS/MS fragment ions used to identify the spectrum during database search, and it also identified assigned annotations for each fragment mass. Peak lists were further analyzed in R. Masses of fragment ions that consistently remained unassigned were extracted separately for biotinylated and nonbiotinylated spectra. Frequencies of unassigned fragment masses were calculated as a fraction of the total number of biotinylated/nonbiotinylated spectra. The resulting frequencies of unassigned fragment masses were used as a screen for diagnostic marker ions of biotin-phenol. Exact masses and chemical structures of the corresponding fragment masses were determined using ChemDraw software.

### Data analysis.

For peptide data, a Spectrum Mill peptide spectrum match report was created. Peptides identified from nonhuman proteins and peptides having a delta forward-reverse score <0 were filtered from the data. For protein data, a Spectrum Mill protein–protein comparison report was created. Nonhuman proteins were filtered from the data. Proteins were also required to have at least two observed SILAC ratios and two distinct peptides in each replicate. For biotin site data, a Spectrum Mill protein-var mod comparison report was created. Biotinylation sites identified from nonhuman proteins were filtered from the data. Using the column “bestDeltaForwardReverseScore,” biotinylation sites with a delta forward-reverse score <0 were filtered from the data. Only fully localized sites were retained by filtering the column “Best_numLocalizedVMsites_y.” Any biotinylation site identified in the SILAC light state was also filtered from the data. Redundant entries were removed by filtering the column “accessionNumber_VMsites_numVMsitesPresent_numVMsitesLocalizedBest_earliestVMsiteAA_latestVMsiteAA” for unique entries.

### Statistical analysis of protein-level-enrichment data.

Statistical analysis of streptavidin-enriched SILAC replicates was carried out as previously described^4^. Our true positive (TP) list consisted of the 173 member list curated in Rhee *et al.*^11^. Our false positive (FP) list was also the same as the one used in Rhee *et al.*^11^, except we removed seven proteins that made it into the Rhee *et al.*^11^ 521 member list. Beginning with Replicate 1 of our current data set, proteins identified by fewer than two unique peptides were eliminated along with known contaminant proteins. Proteins were identified as TP or FP by comparison to the TP and FP lists using gene symbol as the unique identifier. The H/L ratio provided by Spectrum Mill for each detected protein was divided by the median of FP protein H/L ratios. Log_2_ of each resulting value was then taken. This normalization procedure centers the distribution of nonspecific binders around log_2_(H/L) = 0. Next, proteins were ranked in descending order by log_2_(H/L). Receiver operating characteristic (ROC) curves were computed for the sorted members, and this gave a true positive rate (TPR) value and false positive rate (FPR) value for each log_2_(H/L). The cutoff was set as the log_2_(H/L) ratio corresponding to the maximum TPR–FPR value. The above procedure was repeated for Replicates 2 and 3. The three replicates were then crossed, such that only proteins present above cutoff in ≥2 replicates were retained.

### Computational assessment of the surface exposure of tyrosines.

#### Peptides from proteins with crystal structures.

From the APEX experiment, we sought to correlate the experimentally labeled residues with known structural information and thereby ask if the tagged residues correspond with surface accessible residues. A single protein may produce multiple peptides, and a single peptide with more than one tyrosine may have had several tyrosines tagged. All peptides were used as a blast template against the UniProt database^20^, which resulted in 684 unique protein sequences. The resulting parent proteins were then mapped against the PDB database using UniProt’s internal mapping by querying the mapping service (http://www.uniprot.org/help/mapping) with batch scripts. This resulted in 331 unique proteins having 1,759 structures.

The APEX experiment results in covalent adducts being formed with selected tyrosines in a given protein. A protein residue that sits on a surface loop is exposed and likely available for reaction. A residue that sits inside a buried core tertiary structural element is expected to be correspondingly unavailable. While the extremes are straightforward, the overall problem is less so, and several approaches can be considered.

#### Single-residue surface area.

An individual residue’s surface exposure can be estimated by calculating the nonburied molecular surface area of a residue. The APEX experiment results in a reactive radical forming a covalent bond with the aromatic ring of tyrosine. Tyrosyl aromatic rings are often packed edge on against other protein contacts. The reactivity to the reagent might therefore be driven less by backbone exposure than by the hydroxyl exposure or the hydroxyl, ipso, and ortho carbons more than the entire aromatic ring. The exposed surface area of tyrosine was calculated for those atom sets.

#### Crystallized protein state.

Crystallization often requires modification of the protein such as N- or C-terminal truncation or clipping loops, and a simple alignment to the peptide may fail to find the correct tyrosine. Therefore, each peptide was aligned to the canonical UniProt sequence, and both were held fixed while the crystal structure sequence was aligned to this set. The reference tyrosine was then located in the structure and the accessible surface calculated.

Crystal packing complicates an assessment of accessibility. Some packing leads to residues in a buried state that would otherwise be extended into solvent. Other packing is biologically relevant and should not be ignored. These are captured in PDB remark 350 BIOMT transformations. To address these issues, self-homology models of the proteins were created using dissected proteins where the unit cell oligomers were removed but the biomolecular transforms were built after first protonating and charging using MOE’s built-in routine.

#### Aggregation of multiple structures.

Any given protein crystal structure is only one snapshot of the dynamic properties of the protein in solution. We are seeking to correlate a structural element with a cell-derived experimental result. Ideally, we would compute an accurate time-averaged measure of surface exposure. Doing so for the timescale of this experiment is impractical for a single protein. Here, some 1,700 proteins have been identified and therefore need an approximate measure of exposure that leverages all known structure examples. Since crystal packing might artificially constrain a surface residue, the maximum value of exposure was taken from any examples of a protein structure from cases of multiple structures deposited for a given reference sequence.

#### Scoring.

False-negative and false-positive scores were computed. Initially, an absolute cutoff was used, but results were difficult to interpret, especially for false negatives. This is perhaps not surprising, since there can be many reasons for a lack of tagging reaction. The availability of a given tyrosine may be limited structurally within a given protein. It may also protected by protein complexes or protein localization in the cell. Given the difficulty of translating an absolute value across all examples of a protein and under all experimental conditions two approaches were used.

A false negative was scored with an internal reference. Whenever the computed accessible surface area of an untagged tyrosine was significantly greater than the surface area of a tagged tyrosine, the residue was scored as a false negative.

A false positive was declared and scored with a composite value. Visual inspection of many examples suggested an arbitrary threshold of ‘5’ square angstroms. Each such residue was given a score of 10. In addition, a lesser false positive was reported when the value of the tagged tyrosine was less than the smallest value of any untagged tyrosines in a given protein and scored as 1. Thus, a false-positive score of 42 would have four tagged tyrosines with a calculated surface area below 5 and two tagged tyrosines with a surface area above that but less than that of the smallest untagged tyrosine.

A comparison of results from using the self-homology models of biomolecular transform proteins with those from the ‘parent’ crystal structure revealed very few differences in either score and only one for false positives where two additional false positives were reported for 35PA.

The false positives were collated into each detected UniProt sequence and those with a score above 10 scored as the final false-positive set, producing 44 false positives. Inspection of these suggested that most of these cases could adopt an alternate exposed conformation of the side change, which left about 11 as the most likely true false positives.

### Filtering biotinylated peptide data for mitochondrial transmembrane proteins with known topology.

All human IDs used for data annotation were downloaded from Uniprot^20^ FTP (URL: ftp://ftp.uniprot.org/pub/databases/uniprot/current_release/knowledge-base/idmapping/by_organism/, Version: 10/03/2016). The resulting data provided 133,996 unique identifications, which were in turn used to query the Uniprot database. This search returned 109,961 records (including reviewed and unreviewed) along with their functional and structural annotations, including gene ontology features. The annotation table was used to search for mitochondrial evidence associated with a given Uniprot identification in our proximity-labeling anti-biotin-enriched data set by using a string match algorithm. A given protein was annotated as having mitochondrial evidence if any of the following were true: (i) the string ‘mitoch’ matched the above UniProt annotation table when queried by either gene name or accession number. (ii) The protein showed mitochondrial evidence from a list of mitochondrial proteins which was the same as our previous study (Rhee *et al.*^11^) and a precursor to MitoCarta2.0 catalog^28^. (iii) The protein was present in the original mitochondrial true positive list used Rhee *et al.*^11^. Thebiotinylated peptide data were filtered such that reverse hits, nonhuman contaminants, nonfully localized biotinylation sites, sites identified in the SILAC light state, and redundant biotin site entries were removed. From this list, transmembrane proteins were annotated using the following criteria: (i) the UniProt transmembrane annotation contains the string “TRANSMEM” when matched by either gene name or accession number and/or (ii) evidence from of transmembrane domains after running the precursor MitoCarta2.0 catalog through TMHMM^29^. The UniProt topological domain annotation contained the string “TOPO” when matched by either gene name or accession number. From this list of mitochondrial transmembrane proteins, proteins with known topology were extracted by manually inspecting the topology annotation given in UniProt for each protein. The above analysis was repeated for sites identified in ≥2 replicates.

### Data availability statement.

The original mass spectra may be downloaded from MassIVE (http://massive.ucsd.edu) using the identifier MSV000081496. The data is directly accessible via ftp://massive.ucsd.edu/MSV000081496.

A **Life Sciences Reporting Summary** is available.

## Supplementary Material

Supplemental figures and tables

Table S2

Table S3

Table S5

Table S6

Table S7

Table S8

Table S1

## Figures and Tables

**Figure 1 |:**
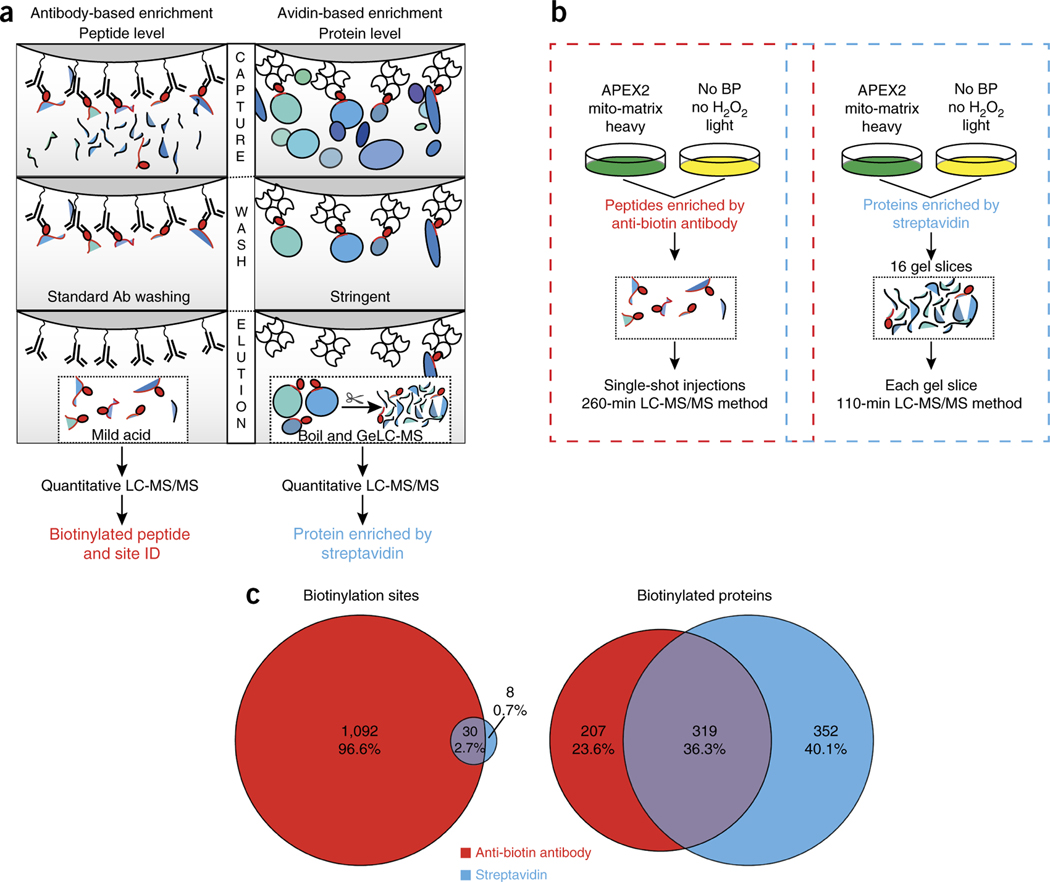
Comparison of antibody-based enrichment and avidin-based enrichment for detection of biotinylated peptides. (**a**) Schematic illustrating the difference in capture, wash, and elution for the identification of biotinylated peptides using either an anti-biotin antibody (left) or an avidin-based workflow (right). The red oval represents biotinylation. (**b**) Schematic illustrating the SILAC-based experimental design used to compare coverage of biotinylated peptides and proteins from proximity labeling samples using either anti-biotin antibodies for peptide enrichment (left) or streptavidin for protein enrichment (right). (**c**) Overlap of biotinylation sites identified using either anti-biotin-based antibody enrichment or streptavidin-based protein enrichment, in ≥2 replicates (left). Overlap of APEX-labeled proteins identified using anti-biotin antibody enrichment or using streptavidin for protein enrichment, in ≥2 replicates (right).

**Figure 2 |:**
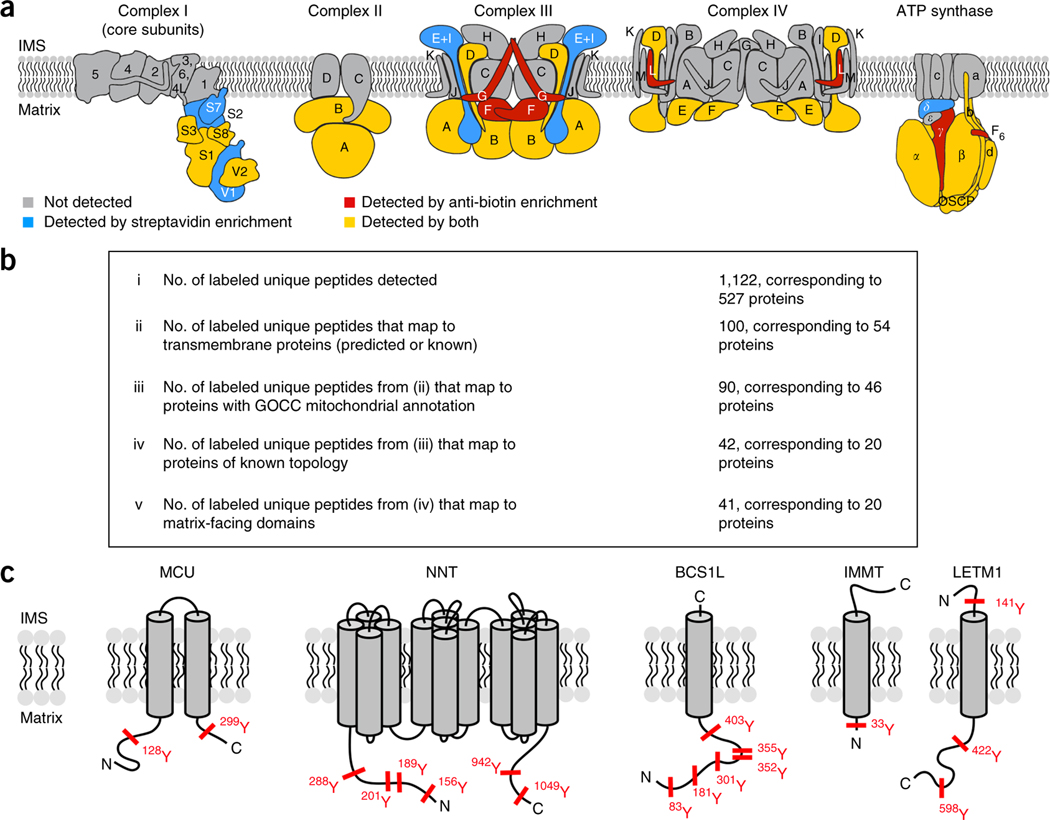
Analysis of APEX2 labeling specificity. (**a**) Comparison of labeling specificity for protein complexes of the IMM. The subunits of complex I (PDB ID: 5LDW), complex II (PDB ID: 1ZOY), complex III (PDB ID: 1L0L), complex IV (PDB ID: 1OCC), and F0-F1 ATP (ATP) synthase are illustrated^24^. Subunits detected above cutoff in ≥2 replicates of the protein-level streptavidin enrichment are shown in blue; subunits with biotinylated peptides in ≥2 replicates of the anti-biotin peptide-level enrichment are colored in red; subunits detected by both are yellow; those not detected are shaded gray. OSCP, oligomycin sensitivity conferral protein. (**b**) Analysis of biotinylated peptides identified using anti-biotin antibody-based enrichment that maps to transmembrane proteins with known or predicted topologies (according to the UniProt database 10/03/16). (**c**) Specific examples of transmembrane proteins from **b** are shown with biotinylation sites depicted in red.
