# Supplementary material for: Antibodies to biotin enable large-scale detection of biotinylation sites on proteins
Source: Nat Methods. Author manuscript; Available in PMC 2022 Mar 25. (PMC8954634; doi:10.1038/nmeth.4465)
Supplement: Supplemental figures and tables [file NIHMS1006645-supplement-Supplemental_figures_and_tables.pdf]

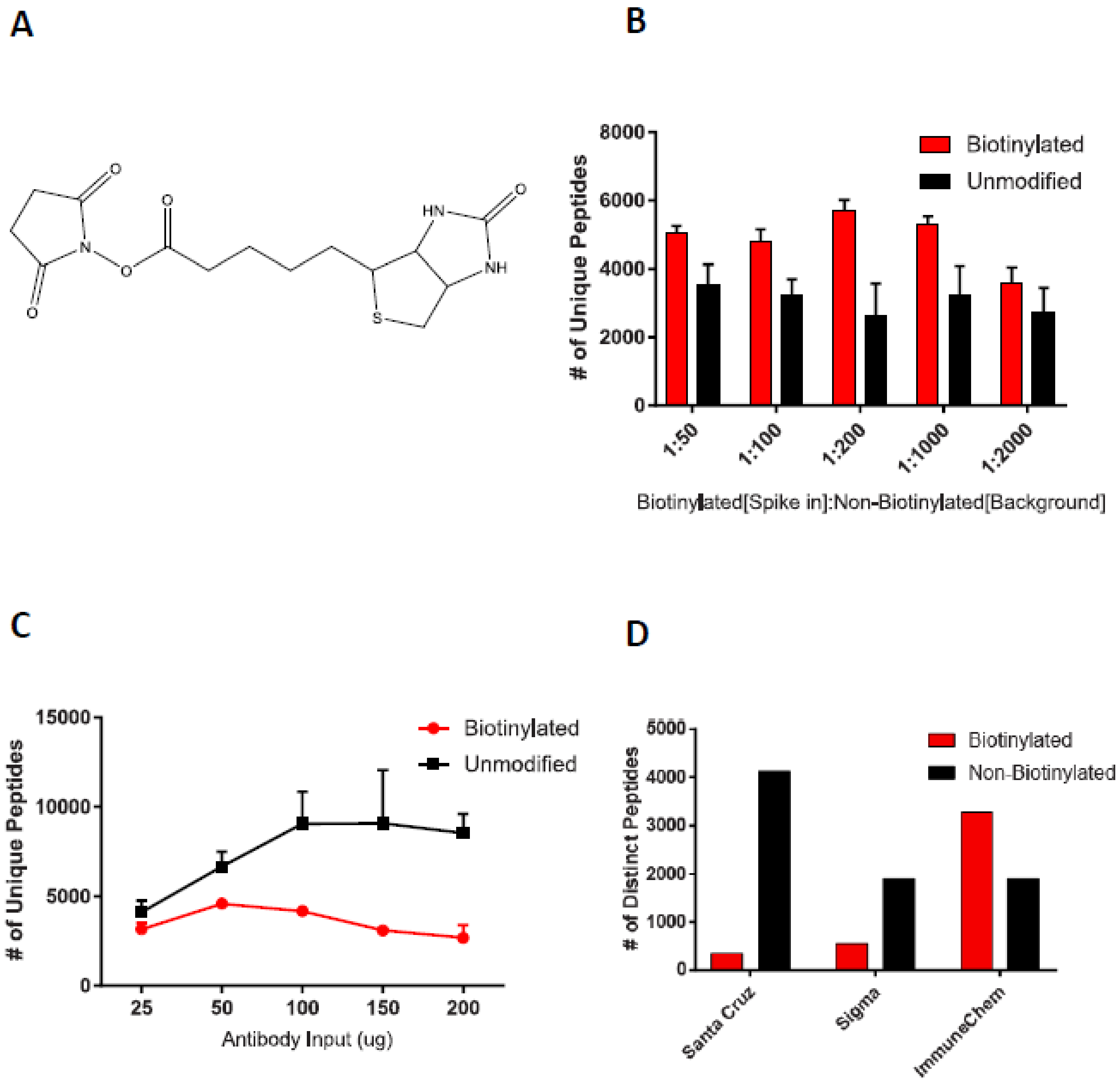

Supplementary Figure 1

Enrichment with anti-biotin antibody significantly increases detection of biotinylated peptides in complex samples.

(A) Structure of NHS-biotin (B) Results of spike-in studies. Bar plot shows the number of unique biotinylated peptides identified at each biotinylated:non-biotinylated peptide spike-in level. Error bars indicate standard deviation across three replicates of enrichment (C) Results of antibody titration experiments. Plot shows the number of distinct biotinylated peptides (red) and the number of distinct non-biotinylated peptides (black) from enrichment of 1 mg of peptides from 1:1000 spike-in samples. Error bars indicate range across two replicates of enrichment. (D) Results of testing different vendor antibodies.

Nature Methods: doi:10.1038/nmeth.4465

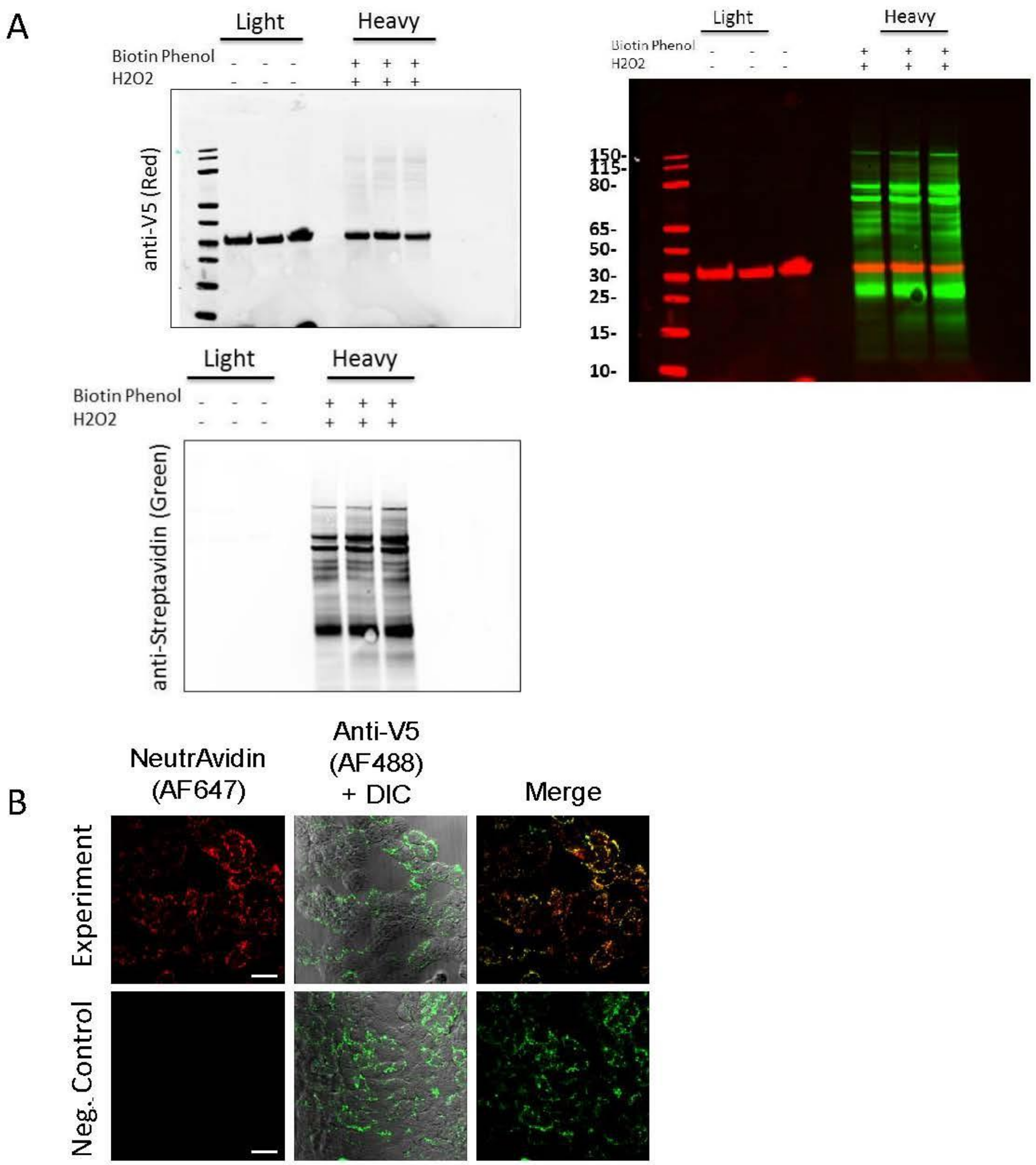

Supplementary Figure 2

Labeling the mitochondrial matrix proteome in living cell.

(A) Anti V5- (top) and and anti-streptavidin (bottom) western blots of SILAC HEK 293T cells labeled with mitochondrial matrix-APEX and biotin phenol. A merged image is also shown. (B) Confocal fluorescence imaging of mitochondrial matrix-APEX2 labeling. Human embryonic kidney (HEK) 293T cells stably expressing matrix-APEX2 were incubated with biotin-phenol (BP) for 30 min and then treated live for 1 min with 1 mM H_2O_2 to initiate biotinylation (top row). A sample in which both BP and H_2O_2 were omitted was prepared in parallel as a negative control (bottom row). Cells were fixed and then stained with a NeutrAvidin-Alexa Fluor 647 (AF647) conjugate to visualize biotinylated proteins and an anti-V5 antibody to visualize matrix-APEX2 localization. DIC, differential interference contrast image. White horizontal scale bars = 25 µm.

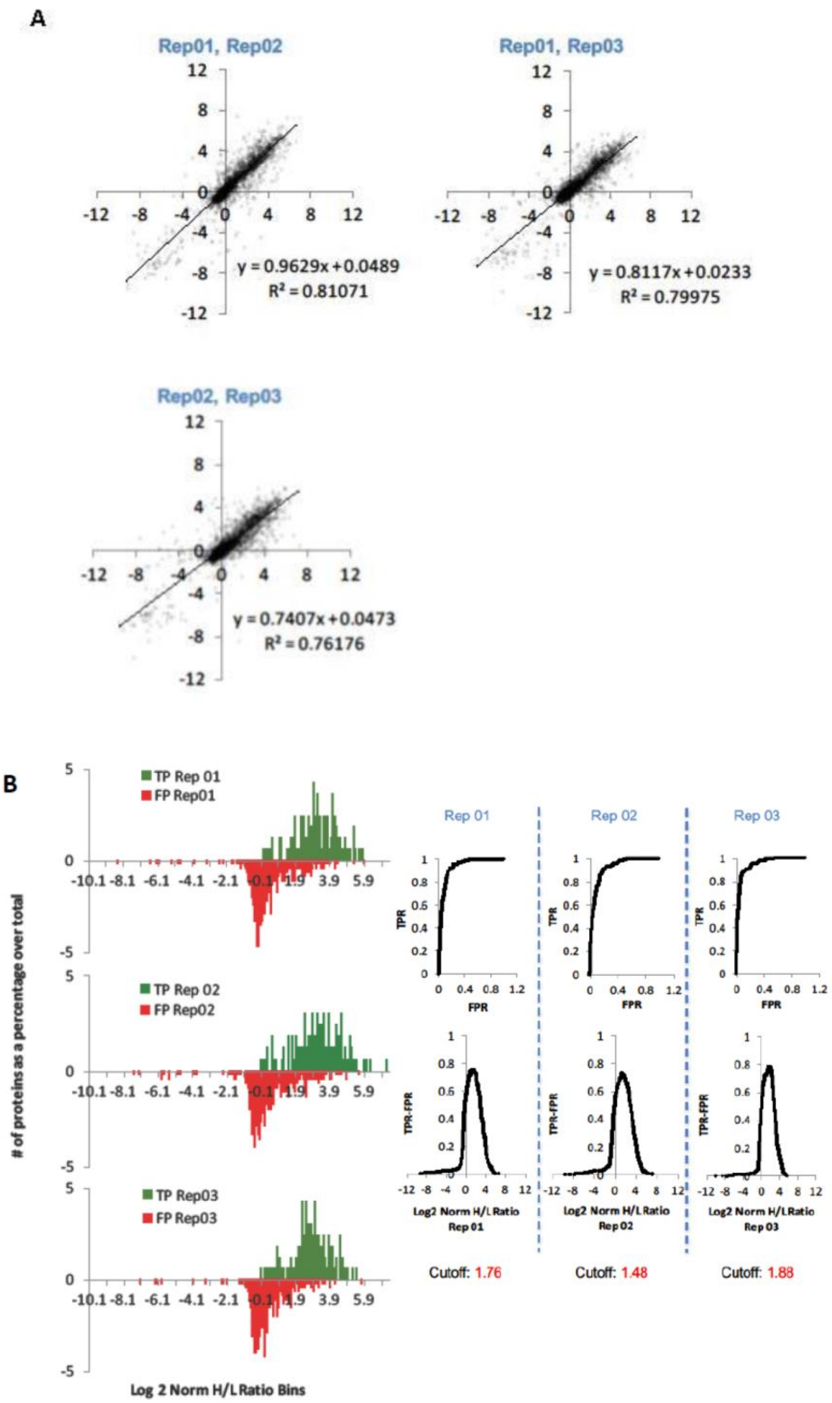

Supplementary Figure 3

Analysis of SILAC data from the mitochondrial matrix streptavidin enriched proteomic experiments.

(A) Correlation between SILAC log2 H/L ratios for replicates 1, 2 and 3. A high correlation is seen, as expected. R^2 values are shown for all detected proteins. (B) Cutoff determination for the mitochondrial matrix streptavidin enriched proteomic experiment. Histograms (left panel) plot true positives (TP) in green, and false positives (FP) in red. Receiver-operating characteristic analysis (ROC, right panel) was performed for each replicate, setting the SILAC ratio cutoff value such that true positives were maximized and false positives were minimized.

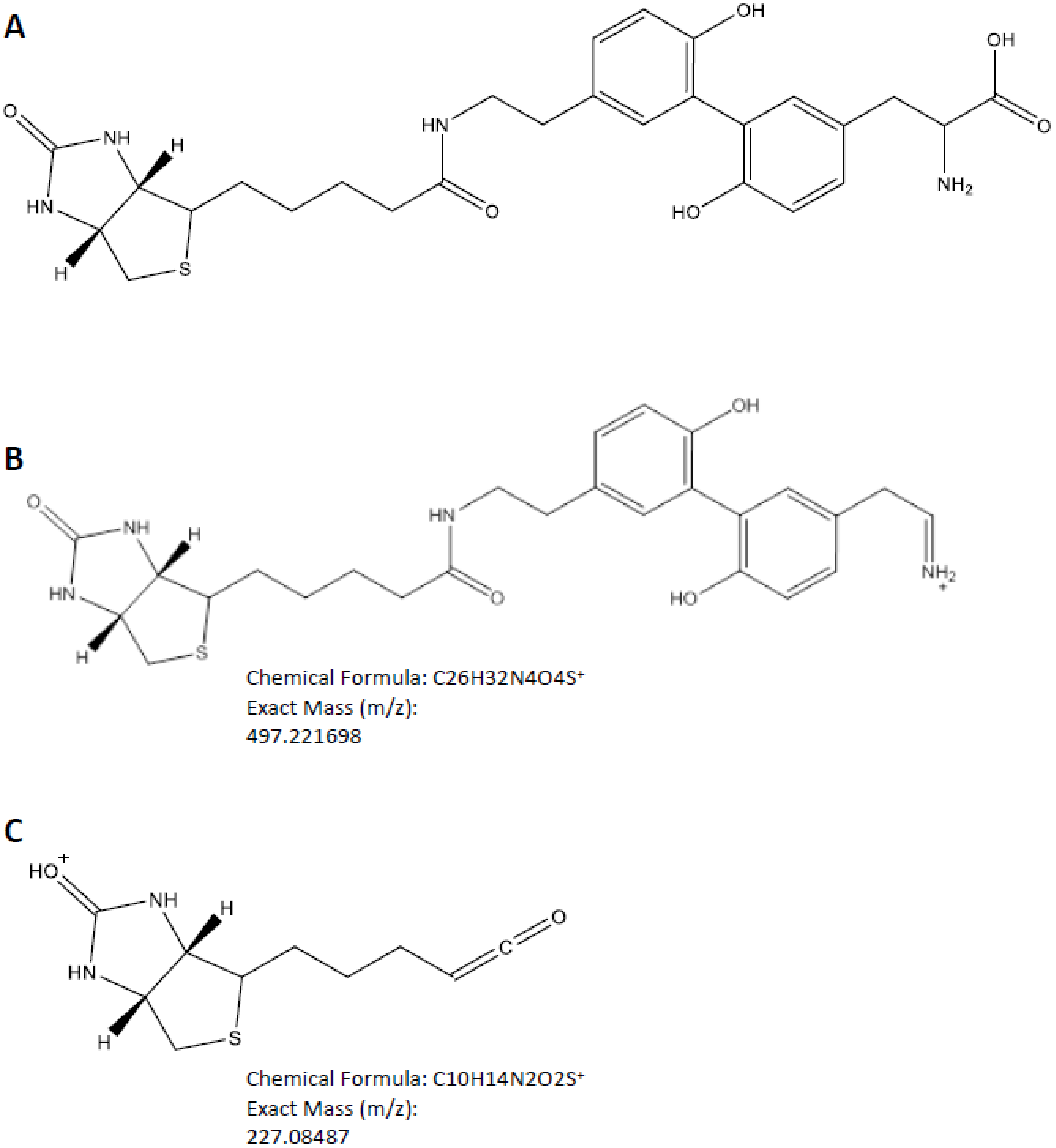

Supplementary Figure 4

Fragment ions specific to biotin phenol can be used to enhance spectral interpretation.

(A) Structure of biotinylated tyrosine (B) Structure and mass of the immonium ion of biotinylated tyrosine (C) Structure and mass of the biotin phenol cleavage product produced upon HCD fragmentation.

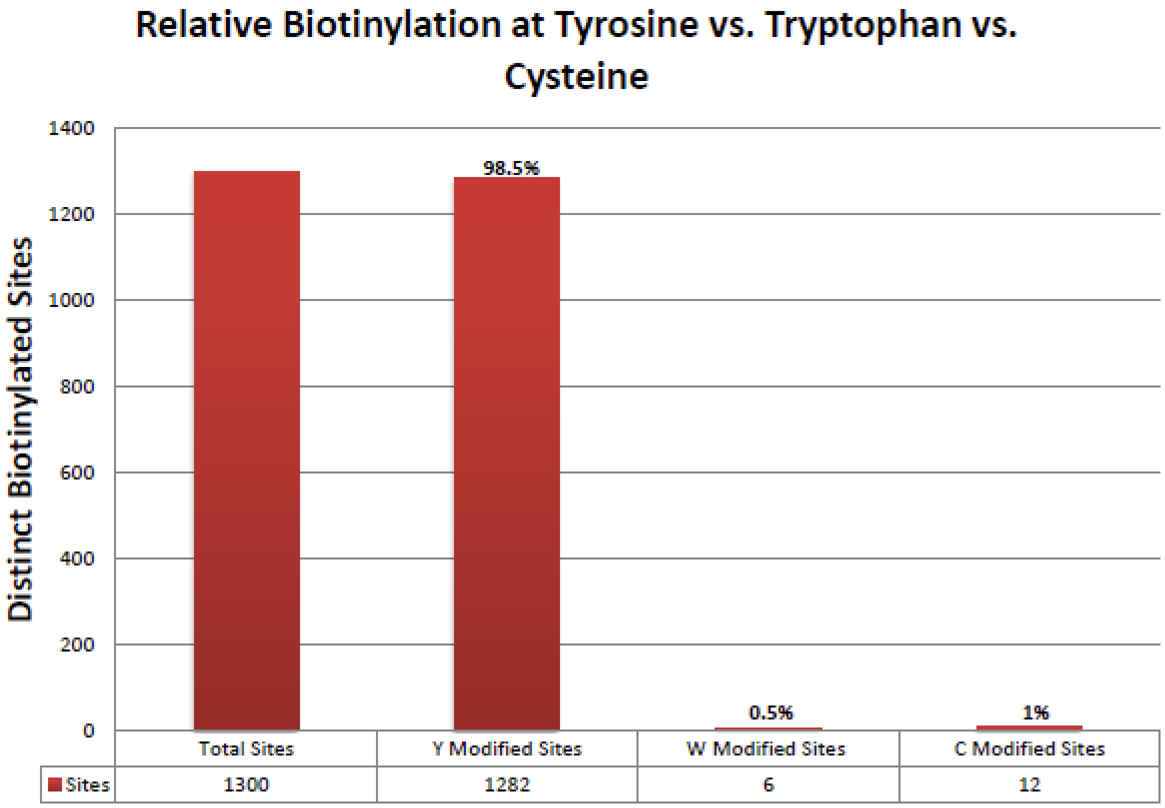

Supplementary Figure 5

Majority of biotinylated peptides identified in antibody enrichment experiments are modified at tyrosine.

Bar plot shows the number of distinct biotinylation sites identified at tyrosine (Y), tryptophan (W), and cysteine (C) from SILAC labeled HEK 293T cells stably expressing matrix-APEX2. The percent of each site at a given amino acid is calculated relative to the total number of biotinylation sites.

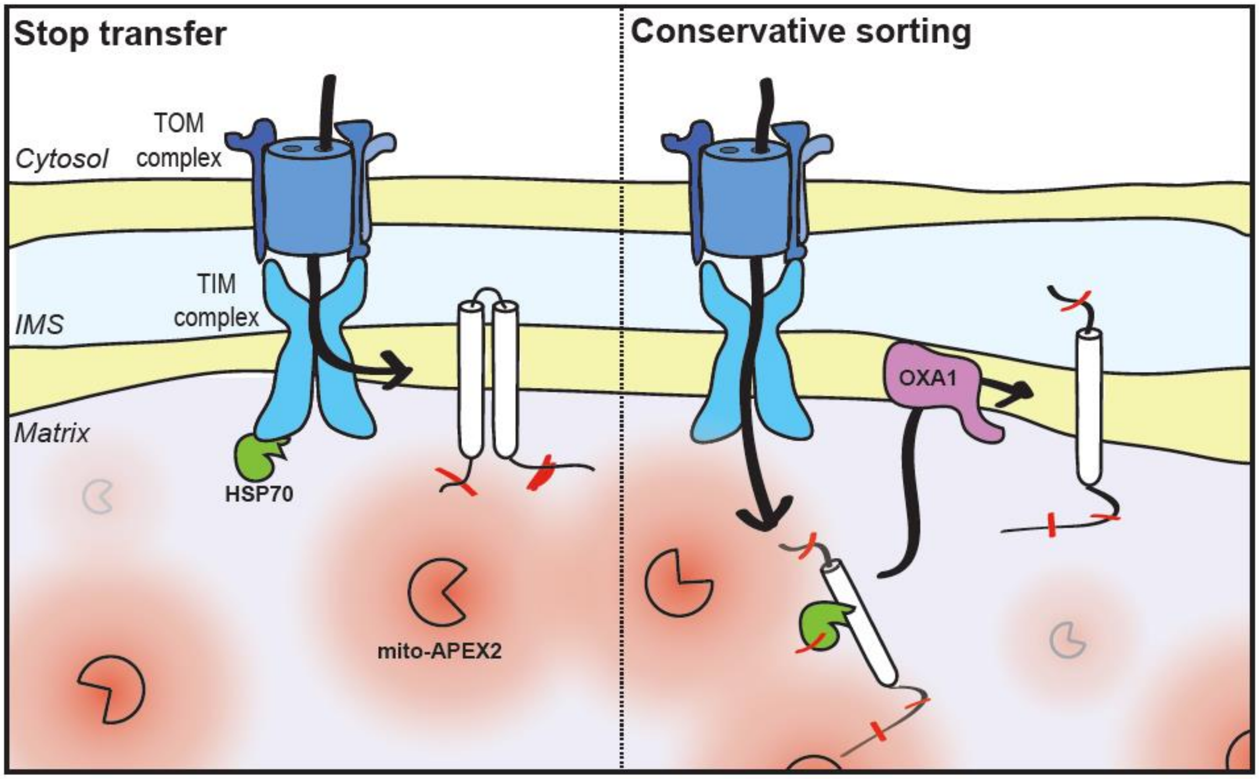

Supplementary Figure 6

Conservative sorting model[21] for LETM1.

(*Left Panel)* Many inner membrane proteins are imported via the Stop-Transfer pathway, where proteins are arrested at the TIM23 complex and laterally instead into the membrane. (*Right Panel*) Another, smaller class of inner membrane proteins are processed via a Conservative Sorting pathway. In this pathway, proteins initially translocate to the matrix prior to insertion in the inner membrane. We propose that biotinylation of Y141 may occur on a processing intermediate of LETM1 if the protein is processed via the Conservative Sorting pathway.

[21]Neupert, W. & Herrmann, J.M. Translocation of proteins into mitochondria. *Annu Rev Biochem* 76, 723-749 (2007).

	Streptavidin Proteome (proteins identified in ≥ 2 replicates)	Streptavidin Proteome (proteins identified in 3 replicates)	Anti-Biotin Antibody Proteome (identified in ≥ 2 replicates)	Anti-Biotin Antibody Proteome (identified in 3 replicates)
**# Proteins**	671	511	526	375
**# Proteins with Mitochondrial Annotation**	473	405	382	303
**# Proteins with no evidence of Mitochondrial Annotation**	198	106	144	72
**% of Proteins with Mitochondrial Annotation**	70.4 %	79.2 %	72.7 %	80.8 %

Supplementary Table 4: Table shows the number of biotinylated proteins identified with either streptavidin protein enrichment or anti-biotin antibody enrichment. For streptavidin-enriched samples, mitochondrial evidence was assigned by crossing our proteome (defined by ROC analysis) with Uniprot, MitoCarta annotations and the true positive list used by Rhee et al[11]. For the anti-biotin antibody-enriched samples, all proteins for which a biotinylation sites was detected were considered and crossed with UniProt annotations, MitoCarta annotations, and the true positive list used by Rhee et al[11].

**[11]Rhee, H.W. et al. Proteomic mapping of mitochondria in living cells via spatially restricted enzymatic tagging. *Science*
339, 1328-1331 (2013).**

	Original Search	m/z 227 480 & 497 Configuration	% Increase
**Replicate 1 # Biotinylated PSMs**	2636	2977	11%
**Replicate 2 # Biotinylated PSMs**	2560	2907	12%
**Replicate 3 # Biotinylated PSMs**	2313	2604	11%

Supplementary Table 9: Diagnostic Ions Improve Identification of Biotinylated Peptides. Table presents the number of biotin phenol modified peptides identified in each biotin enrichment replicate. Results are shown for data searched with and without diagnostic ions (specific to biotin and biotin phenol) configured in Spectrum Mill.
